# The meningeal–cerebellar axis: a new perspective on cerebellar development

**DOI:** 10.1007/s00018-025-05897-1

**Published:** 2025-12-02

**Authors:** Amnah Al-Sayyar, Laure Salvon, Narjess Haidar, Paul Schult, Oussama Kassem, Rejane Rua, Audrey Romano

**Affiliations:** Aix-Marseille Université, CNRS, INSERM, Centre d’Immunologie de Marseille-Luminy, Marseille, France

**Keywords:** Meninges, Cerebellum, Development, Neuroimmune, Cytokines

## Abstract

The cerebellum is a highly organized brain structure best known for its roles in motor control and sensorimotor integration. While cerebellar development has traditionally been attributed to intrinsic genetic programs and local cell–cell interactions, emerging evidence indicates that extrinsic cues particularly signals from the meninges also play a critical role in shaping its maturation. Studies indicate that the meninges release cytokines, chemokines, and growth factors including CXCL12, IGF-1, IL-33, FGF2, TGF-β, and retinoic acid that influence granule cell precursor (GCPs) proliferation, Purkinje cell (PC) maturation, radial glia organization, and synaptic refinement. In addition, meningeal immune cells form a dynamic interface that potentially shapes neuronal positioning and cerebellar circuit formation. Disruption of these signals through genetic mutations, immune dysregulation, or environmental insults lead to impaired foliation, ectopic neuronal migration, and aberrant cerebellar architecture. This review focuses on in vivo findings supporting an emerging concept of the meningeal-cerebellar axis in development. Understanding cerebellar maturation within this broader context offers new perspectives on the origins of neurodevelopmental disorders and points toward novel avenues for therapeutic intervention.

## Introduction

The concept of the central nervous system (CNS) being immune privileged was historically debated. This stemmed from observing that systemic immune responses to tumors, viruses and bacteria were often absent in the brain parenchyma [1]. As interest grew in this field, evidence suggest that the brain and immune system interact through extensive bidirectional communication that involves the release of multiple neurotransmitters, cytokines and trophic factors that act as mediators in health and disease [2–4]. CNS barriers such as the choroid plexus, meninges and blood-cerebrospinal fluid (CSF) represent niches for immune cells creating an environment that helps sustain healthy brain development, function and structural integrity [5–7]. Immune cells residing at these interfaces play essential roles in neurodevelopment, and their dysregulation has been implicated in the pathogenesis of neurodevelopmental disorders such as autism spectrum disorder (ASD) and schizophrenia [7].

### The meninges

The meninges are composed of three distinct layers (the dura mater, arachnoid mater and pia mater) which envelop and isolate the CNS from the rest of the body [8]. This structure serves as a critical interface between the peripheral immune system and the brain, playing a key role in immunosurveillance during both development and homeostasis [8]. The outermost layer, the dura mater lies adjacent to the skull and contains meningeal lymphatic vessels that communicate with the glymphatic system to regulate waste clearance and immune cell trafficking [8]. Additionally, dural cells release neuromodulators such as noradrenaline, acetylcholine, and neuropeptides, influencing local neural and immune environment. [8]. The inner layer consists of the arachnoid and pia mater which are separated by the subarachnoid space forming the leptomeninges [8]. Epithelial-like cells in the arachnoid mater are connected by tight junctions forming adjacently to the subarachnoid space where the CSF circulates [8]. Furthermore, the pia mater follows the contours of the brain and spinal cord and consists of a monolayer cell composed of the parenchymal basement membrane and astrocyte end-feet [8].

#### Meningeal cells

The meninges are a highly dynamic immunological tissue in which a wide repertoire of resident and migratory immune cells are located [9]. Several single-cell transcriptomic and spatial mapping studies revealed layer-specific immune niches within the dura, arachnoid, and pia mater [10, 11]. In particular, the dura houses most of the meningeal immune cells (both innate and adaptive cells) including macrophages, dendritic cells, mast cells, T and B cells and innate lymphoid cells (ILCs) due to its close proximity to the skull bone marrow and close association with the dural venous sinuses [10–12].

Border-associated macrophages (BAMs) are specialized macrophages that reside at the interfaces of the CNS. They originate from the yolk sac during embryogenesis, with additional contribution from adult hematopoietic cells to the dura mater and choroid plexus [13]. Even though microglia and BAMs are derived from the same yolk sac progenitors, single cell analysis have shown that they are transcriptionally distinct, and BAMs exhibit regional heterogeneity depending on their precise location (dura vs. leptomeninges vs. perivascular regions) [13]. BAMs play a role in regulating immune surveillance, pathogen response and waste clearance [13]. Several reviews highlighted the distribution and functions of natural killer (NK) and ILCs in CNS border regions at steady state in adulthood, and their roles in major neuroinflammatory and neurodegenerative conditions [14, 15]. NK and ILC1 cells residing in the dura mater contribute to brain homeostasis, regulate neuronal circuitry and neurotransmission, and influence glial activity through the release of cytokines and cytotoxic mediators, thereby participating in anti-tumor immune responses against glioma [16–18]. Moreover, T cells, including tissue-resident memory subsets, are mainly found in the meninges and choroid plexus at steady-state [19]. Beyond their role in immune surveillance, their presence is implicated in modulating several CNS physiological processes such as social behavior, anxiety, learning, and memory [20]. The functions of distinct meningeal T cell subsets under homeostatic conditions have been extensively described [21]. Notably, T cell–derived IFN-γ regulate social behavior and inhibitory circuits located closely to the brain surface and the CSF, while conditional deletion of IFNγ receptors induced social behavioral deficits similar to mice lacking mature T cells [22]. In addition, the absence of CD4⁺ T cells impairs inhibitory synaptic function and disrupts long-term potentiation, leading to memory deficits [23]. CD8⁺ and Th17 cells are less abundant under steady-state conditions but contribute to CNS immune surveillance and maintenance of tissue integrity [21, 24]. Interestingly, meningeal γδ T cells are present during early development and play a pivotal role in regulating anxiety-like behavior through IL-17A signaling [19, 25]. These findings suggest that meningeal T cells-mediated cytokine release contributes to neuronal circuit functions and related behaviors.

A recent discovery of functional lymphatic vessels in the dura has reshaped the understanding of immune cell drainage and antigen transport to cervical lymph nodes, establishing the meninges as an active immunological checkpoint [26]. Other than immunes cells, meningeal fibroblasts play an important role in maintaining the integrity of the meninges [27]. They also secrete a range of signalling molecules that influence CNS development, vascularization and homeostasis [27]. Single cell transcriptomic analyses showed the heterogeneity of meningeal fibroblasts populations embryonically, with distinct subpopulations distributed across different meningeal layers [27]. These findings underscore the meninges as a dynamic and immunologically active interface, where diverse resident immune and stromal cell populations interact to support CNS function and potentially influence its development.

#### Development of the meninges

The development of the meninges is closely coordinated with CNS maturation [28]. It is formed during early embryogenesis, when mesenchymal cells envelop the hindbrain, midbrain, and forebrain at the time of neural tube closure [29]. At this stage (E10 in mice; 4th week of gestation in humans), the meningeal layers are not yet distinct but contain vascular plexus along the brain surface, which later differentiate into blood vessels supplying both the meninges and the brain [29]. In parallel, neural stem cells undergo mitotic divisions that generate radial glial precursors, and the first immature yolk sac–derived macrophages appear in the developing CNS [28]. By E12–E13 in mice (~ 6th gestational week in humans), the meninges are organized into two primary layers: the external dermal layer and the calvarial layer, the latter containing precursors of sutures and bone [29]. Beneath the calvarial layer, the meninges are further subdivided by the dural limiting layer into the dura mater and the leptomeninges [29]. During this period, meningeal fibroblasts begin to express layer-specific markers in a ventral-to-dorsal gradient, reflecting spatial maturation [28]. These developmental windows are also characterized by intense mitotic activity, cellular migration, angiogenesis, and vessel growth, orchestrated by multiple developmental signaling pathways including Bone Morphogenetic Protein (BMP) pathway [28]. By E14 in mice (~ 10th gestational week in humans), the meningeal layers are established, with the exception of the arachnoid space, which continues to develop until birth [28]. Completion of arachnoid morphogenesis is dependent on the maturation of the blood–CSF barrier, which itself is linked to the blood–brain barrier (BBB) formation (starts at E13-E15), both of which reach full maturation postnatally [28]. Additionally, radial glial precursors differentiate into oligodendrocytes and astrocytes, while the macrophage populations become more defined, giving rise to CD206⁺ BAMs and microglia [28].

Postnatally (P0–P21 in mice; approximately the 24th gestational week to 1 month postnatal in humans), the dura layer thickens as it becomes enriched with collagen fibers [28, 29]. During this period, meningeal lymphatic vessels mature in parallel with the glymphatic system, and BAMs (CD206⁺/Lyve1⁺) can be distinguished from microglia (P2Y12⁺/Sall1⁺) [28]. In addition, the sagittal and coronal sutures are established and populated with osteoblasts that support cranial bone renewal, coinciding with ongoing gliogenesis, myelination, and synaptic development and refinement within the neocortex [28]. These developmental milestones highlight the meninges as a dynamic and evolving structure that not only provides mechanical protection but also actively coordinates vascular, immune, and neuroglial maturation during critical windows of CNS development.

### The cerebellum

The cerebellum contains the majority of the brain's neurons, accounting for approximately 60% in mice and up to 80% in humans [30]. Traditionally, it has always been viewed as a motor structure that is important for movement coordination, posture, and speech. However, due to its connectivity to different brain regions, its role expanded to include non-motor functions such as cognition, emotions modulation, attention, decision making and appetite regulation [31–37]. The cerebellum’s participation in higher-order functions was highlighted in the late 1990s, where cerebellar lesions in patients caused deficits in executive functions, visuospatial organization, cognition and linguistic abilities; which was defined collectively as Cerebellar Cognitive Affective Syndrome (CCAS) [38, 39]. Abnormal cerebellar functions are linked to key risk factors such as genetic predisposition, neurodevelopmental damage, and neuroinflammation which contribute to the progression of various neuropsychiatric and neurodevelopmental disorders [31]. This association stems from the cerebellum's interplay between associative learning and motor adaptation functionality that influences cognition and emotional regulation, occurring specifically between the Deep Cerebellar Nuclei (DCN) and the cerebral cortex [31]. As a result, cerebellar dysfunction can disrupt a broad spectrum of cellular, morphological, and neural circuit networks [31].

#### Morphology and development

**Fig. 1:**
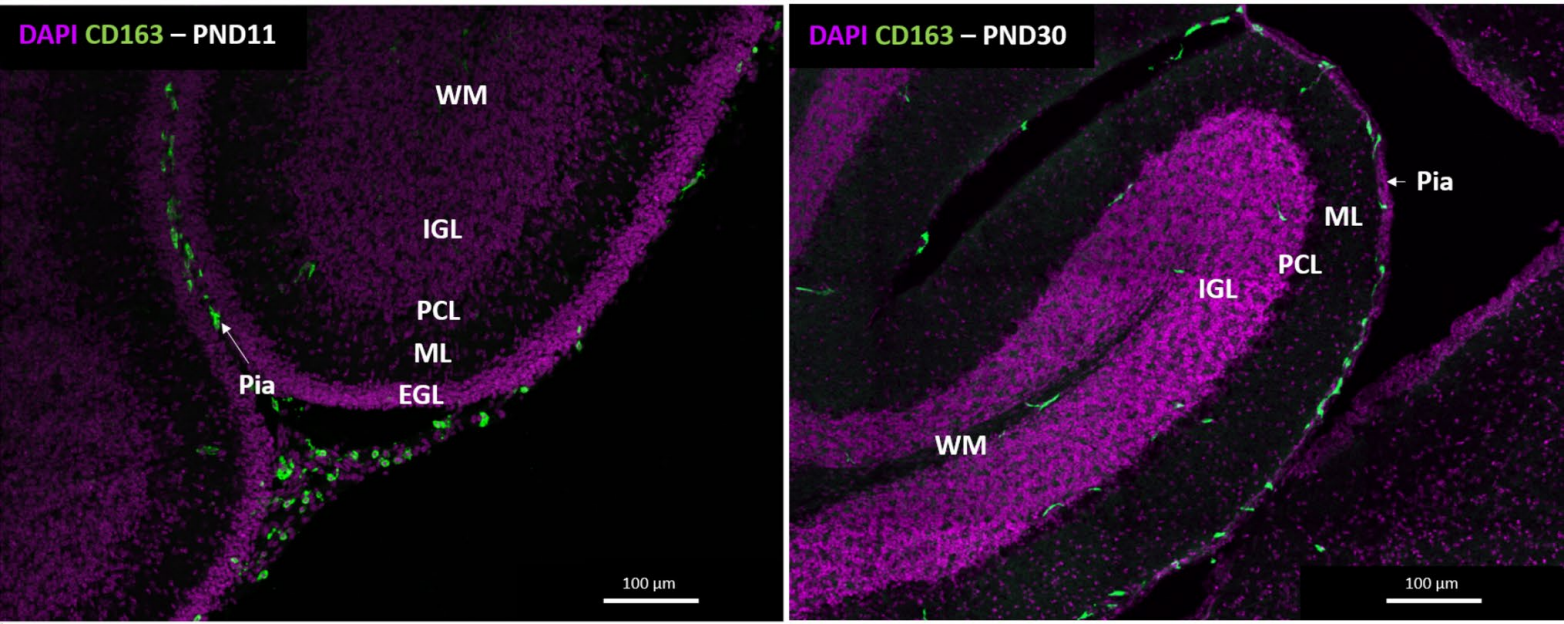
Localization of CD163⁺ meningeal macrophages along the cerebellar surface during development. Representative sagittal cerebellar sections from postnatal day (PND) 11 and PND30 mice stained with DAPI (nuclei, magenta) and CD163 (meningeal macrophages, green). Cerebellar layers are indicated: external granular layer (EGL), molecular layer (ML), Purkinje cell layer (PCL), internal granular layer (IGL), and white matter (WM). At PND11, CD163⁺ macrophages align with the pia mater adjacent to the EGL, following the contours of developing folia. By PND30, CD163⁺ cells remain localized along the pia and blood vessels, delineating the meningeal–cerebellar interface. Scale bars = 100 μm

Compared to other brain regions, the cerebellum is distinguished by its intricate architecture of 10 main folds known as lobules which are separated by fissures that define the folds depth [40]. Each foliation is comprised of three layers that house unique neuronal cell populations. The outermost layer located directly under the pia mater is known as the molecular layer that contains inhibitory stellate and basket cell interneurons, in addition to excitatory climbing fibers (CF). All three pass through the Purkinje cell layer (PCL) and project directly to the Purkinje cells (PCs), which are the sole inhibitory output neurons of the cerebellum. The last and deepest layer is known as the granule cell layer which consists of excitatory granule cells (GCs), inhibitory Golgi cell interneurons, excitatory mossy fibers (MF) and unipolar brush cells [40, 41]. In addition, GCs extend their axons to the molecular layer forming parallel fibers (PF) that provide excitatory input into PCs [31]. Below the three layers is the cerebellar white matter where the DCN is located in addition to specialized neurons that transmit the final output of the cerebellum linking it to the rest of the brain and spinal cord (Fig. 1) [40].

The development of the cerebellum is a highly orchestrated process that begins around E13 to P20 in mice and from the 7th gestational week to one year postnatally in humans [31]. This suggests that cerebellar maturation has a broader developmental window which makes it susceptible to various risk factors and disorders as it is the first brain structure to start cell proliferation and the last to mature [31]. The development process occurs in four stages; 1) defining the cerebellum territory through genetic regulation; 2) cellular differentiation and proliferation of PCs and granular cell precursors (GCPs); 3) Transition of the External Granular Layer (EGL) to Internal Granular Layer (IGL) through the inward migration of the GCs, supporting cerebellar expansion; and 4) neuronal circuit formation and further differentiation [42] (Fig 1). These processes are controlled by several developmental signaling pathways such as Sonic hedgehog (Shh), Wingless-nt (Wnt), Fibroblast growth factors (Fgfs) and Brain Derived Neurotrophic Factor (BDNF) which are precisely timed and spatially controlled to shape the cerebellum’s morphology, connectivity, and overall architecture [31, 40]

**Table 1:** Summary meningeal-cerebellar factors in development (Wild-type and reporter mouse models)

Factor	Type	Genotype	Sacrifice timepoint	Readout	References
CXCL12	Ligand	Reporter: Col1a1-GFP^GFP/+^	Embryonic (E14)	CXCL12 expression in Meningeal Fibroblasts	[27, 55]
CXCR4	Surface Receptor	Reporter: SDF-1:mRFP/CXCR4-GFP	Embryonic (E12-15)	CXCR4 expression in radial glia end-feet in the pia matter	[55]
FGF2	Growth Factor	Wild-type CD1 mice	Embryonic: E12.5 and E16.5 Postnatal: P0, P11 and P21	FGF2 is markedly upregulated in the cerebellum at P1, with its expression being precisely regulated in coordination with key developmental milestones, including GCPs in the EGL at P0, GCs in the IGL at P7, and PCs in the PCL by P21	[67]
		Wild-type Sprague–Dawley rats	Postnatal: P1, P3, P7, P14, P21 and P28	FGF2 expression is at its lowest at postnatal week 1 and highest by postnatal day 28	[68]
IL-33	Ligand	Wild-type C57Bl/6 mice to study spatiotemporal expression of IL-33	Embryonic: E11, E13, E15, E17, E19 Postnatal: P2, P9, P16, P23	IL-33 is highly expressed in the cerebellum at P9	[81]
		Reporter: Il-33–LacZ gene trap (Gt) reporter strain (Il-33^Gt/Gt^)	Embryonic: E15.5	IL-33 is mainly localized in cerebellar GCs and white matter layers	[83]
Retinoic Acid	Ligand	Reporter: RARE-hsplacZ	Embryonic (E16, E17) Postnatal (P3)	RA is expressed in cerebellar and pre-cerebellar regions	[88]
TGF-β1	Ligand	Wild-type CD1 embryos	Embryonic: 11.5, E13.5, E15.5, E18.5	TGF-β1 highly expressed at E11.5 in the cerebellum and is expressed along with pathways linked to cell–cell interaction, migration and neurons placement	[99]

**Table 2:** Summary meningeal-cerebellar factors in development (In vivo mouse models)

Factor	Type	Genotype	Sacrifice timepoint	Readout	References
SDF-1/CXCL12	Ligand	Full-knockout: SDF-1^−/−^	Embryonic (E18.5)	Impaired GCPs migration and proliferation and ectopic PCs localization	[57]
CXCR4	Surface Receptor	Full-knockout: CXCR4^−/−^	Embryonic (E13.5–18.5) Postnatal day 0	Impaired GCPs migration and proliferation and ectopic PCs localization	[56] [57]
		Full-knockout: CXCR4^−/−^	Embryonic (E17 and E19)	Impaired GCPS and PN neuron migration	[58]
		Conditional knockout: Nestin^Cre^;Cxcr4^LoxP/LoxP^	Embryonic (E13.5, E14.5, E15.5, E17.5, E19.5)	Impaired GCPs migration and BGs and PCs localization	[59]
		Conditional knockout: Sox1^cre^;CXCR4^flox/flox^	Postnatal (PND1-PND9)	Impaired PCs dendrites and cell–cell adhesion pathways	[62]
Foxc1	Upstream transcription factor of SDF-1/CXCL12	Full-knockout: Foxc1-/-	Embryonic (E13.5, E14.5, E15.5, E17.5, E19.5)	Impaired GCPs migration and BGs and PCs localization	[59]
		Hypomorphic: Foxc1^hith/hith^	Embryonic (E14.5, E15.5, E17.5) Postnatal (P0, P1, P15, P21)	Impaired GCPs migration, cerebellar foliations and lamination	[60]
Pdgf-c	Growth factor	Full-knockout: Pdgfc^−/−^;Pdgfra^GFP/+^ (Cerebellar hypoplasia model)	Embryonic (E11.5, E12.5, E14.5, E15.5, E17.5) Postnatal (P0, P1, P2, P4, P15, P19,)	Missing lobules, irregular pattern formation, downregulation of genes linked to PCs (Calb1) and meninges (CXCL12, Crym)	[61]
Fgfr1/2	Receptor	Double Knockout: hGFAP^Cre^;Fgfr1^f/f^;Fgfr2^f/f^	Embryonic: E16.5 Postnatal: P7 and P14	Impaired GCs migration and proliferation, cerebellar morphology, PC and BG location	[69]
		Conditional Knockout: Nestin^Cre^;Fgfr2^lox/lox^	Embryonic: E14.5, E16.5, E18.5	Impaired BG localization and reduced BG number along with reduced FGF signaling	[70]
IGF-1R	Receptor	Conditional Knockout: Math1cre;Igf-1r^flx/flx^	Postnatal: P21	Reduction in cerebellar weight and reduced GCs in anterior foliations only (II – VI)	[77]
		Conditional Knockout: MADM-Igf-1r	Postnatal: P21	Reduced GCs in anterior and posterior foliations along with impaired proliferation (premature cell cycle exit)	[77]
IGF-1	Ligand	Medulloblastoma mouse model: Ptc^±^;Igf1-Tg: (overexpression IGF1 in neural progenitors)	Postnatal: P5, P15, P21	Impaired cerebellar morphology and GCPs over-proliferation	[79]
IL-33/Il1RL1	Ligand/Receptor	Conditional Knockouts: GFAP^cre^;IL-33^flx/flx^ Cx3cr1^cre^; Il1RL1^flx/flx^	Postnatal: P9, P28	Microglial dystrophy, impaired synaptic function, and behavioral abnormalities	[85]
RORα	Nuclear Receptor	Conditional knockout: Pcp2^cre^;Rorα^flx/flx^	Postnatal (P14)	Impaired PCs morphology and connectivity	[90]
		Conditional knockdown: miR-RORα + EGFP (In utero electroporation into precursor PCs at E11.5)	Postnatal (P8, P14, P21)	Impaired PCs dendritic maturation and branches; increased axons swelling	[91]
		Overexpression: L7-CreERT2, 4-OHT–inducible expression of rRORα1-HA (Purkinje cell-specific, tamoxifen-inducible transgene expression) at P4	Postnatal (P13)	Accelerated PCs maturation at the fusiform stage	[91]
Smad2	Transcription Factor (Downstream TGF-β1)	Conditional knockout: Nestin^Cre^;Smad2^flox/flox^	Embryonic and Postnatal (E16.5-P19)	Impaired GCs migration and maturation, PCs dendrites, and overall cerebellar development	[100]
Smad3	Transcription Factor (Downstream TGF-β1)	Partial Knockout: Ptch ^±^ (knockout of Shh receptor)	Postnatal: P1, P7, P13, P20	Impairs GCPs differentiation and migration	[101]
Wnt5a	Ligand	Conditional Knockout: Nestincre;Wnt5aflox^/flox^	Embryonic: E14.5, E18.5 Postnatal: P1, P7. P14	Impaired cerebellum morphology, and PCs and GCPs neurogenesis	[103]
Tmem67	Transmembrane protein	Full knockout: Tmem67^−/−^	Embryonic: E12.5, E15.5 E18.5 Postnatal: P0, P1	Impaired cerebellum morphology and developmental signaling (Shh an Wnt/βcatanin)	[104]
Laminin-γ1	ECM Protein	Full knockout: γ1III4^−/−^	Embryonic: E10.5, E12.5, E13.5, E16.5, E17.5	Impaired pial basement membrane morphology and ectopic neuron migration	[106]
Laminin- α1	ECM Protein	Conditional Knockout: Sox2^cre/+^; Lama1^flox/del^	Postnatal: P0, P5, P10	Impaired pial basement membrane, cerebellar morphology and BGs alignment	[105, 107]
ILK	Scaffold Protein	Conditional Knockout: gfap^Cre^; ilk^loxP/loxP^ or Nestin^Cre^; ilk^loxP/loxP^	Postnatal: P10	Defective laminin deposition, abnormal glial morphology, and alterations in GCs migration	[108]

While studies on cerebellum development mainly focus on neuronal specification, proliferation and migration, it is important also to highlight the final stage of cerebellar circuit formation as it heavily depends on synaptic refinement process. Microglia are critically involved in neural circuit refinement by mediating synaptic pruning [43]. During cerebellar development, multiple CF innervate the PCs soma which are gradually eliminated to attain mono-innervation (i.e. one CF projects into the PCs) [44]. Interestingly, suppressing GCPs proliferation by applying X-ray irradiation during the first development week (postnatal day 4–7) impaired PF input into the PCs that affected CF mono-innervation [45]. This highlights that, GCs are essential for effective synaptic elimination which is important for fine-tuning neuronal circuitry to support the cerebellum’s roles in motor coordination, timing, and adaptive learning [31].

**Fig. 2:**
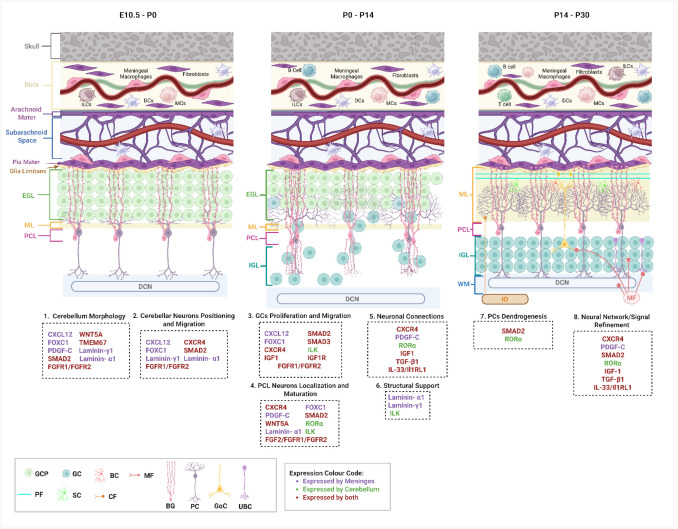
Immune–meningeal interactions supporting cerebellar development – Focus on in vivo mouse models. This schematic illustrates the dynamic interactions between meningeal cells, immune populations, and cerebellar neurons across key developmental stages E10.5–P0, P0–P14, and P14–P30. Meningeal layers (dura, arachnoid, pia) and their resident immune cells including meningeal macrophages, dendritic cells (DCs), mast cells (MCs), innate lymphoid cells (ILCs), B cells, and T cells are shown at the first layer. The subarachnoid space is traversed by vasculature and bounded by the glia limitans at the pial surface, forming a critical interface with the underlying cerebellar cortex. The cerebellum includes the external granular layer (EGL), molecular layer (ML), Purkinje cell layer (PCL), internal granular layer (IGL), and white matter (WM) with deep cerebellar nuclei (DCN). Color-coded genes indicate expression in meningeal cells (purple), cerebellar cells (green), or both (red). Developmental processes are annotated at the bottom of each panel and linked to key signaling molecules that coordinate meningeal-cerebellar interactions during different developmental stages. (Created in BioRender. Alsayyar, A. (2025) https://BioRender.com/xb4ntde)

Beyond their traditional role in immune surveillance, the meninges invaginate the cerebellum’s hemispheres and foliations contributing to its development (Fig. 1) [46]. The meningeal-cerebellar interaction is mediated by a diverse array of cytokines, chemokines and growth factors that influence key developmental processes including neuronal migration proliferation and synaptogenesis (Fig. 2 and Tables 1, 2 and 3) [47–49]. This review will provide a foundation on the multifaceted interactions between the meninges and the developing cerebellum, with a particular emphasis on key factors such as cytokines, chemokines, and growth factors that originate from the meningeal layers using several mouse models (Table 4). By integrating findings from both classical and recent experimental models, we aim to shed light on how these signaling molecules influence cerebellar patterning, cellular differentiation, and synaptic maturation. Understanding this meningeal-cerebellar axis not only broadens our view of neurodevelopmental regulation but also provides novel perspectives on the etiology of cerebellum-linked neurodevelopmental disorders and potential avenues for therapeutic intervention.

**Table 3:** Summary meningeal-cerebellar neuroimmune factors in development (Treatment and *ex-vivo* experiments)

Factor	Type	Method	Sacrifice timepoint	Readout	References
FGF2	Growth Factor	*Ex-vivo*: Isolation of CD44 + or CD44- cells from P3 cerebellum of ICR mice	Postnatal: P3	FGF2 promotes the differentiation of oligodendrocytes progenitors	[71]
IGF-1	Ligand	Exogenous administration of IGF-1 using a series of doses into a denervated cerebellum after unilateral pedunculotomy	Postnatal: P11, P12, P15, P20, P30	Enhanced myelination, neuroplasticity and innervation between PC-CF	[78]
RORα	Receptor	Intra-peritoneal injection of VPA 500 mg/kg body weight dissolved in PBS (10.5 days post coitum E10.5) – Model of ASD	Postnatal: P1, P10, P30	Reduced RORα, impaired PCs synaptic connectivity, long-term behavioral deficits	[92]
Retinoic Acid	Ligand	Exogenous treatment: 250 mg/kg weight SC injection of all-trans RA agonist	Postnatal (P14, P30)	Impaired GCs proliferation and migration	[93]
TGF-β1	Ligand	*Ex-vivo*: isolation of cerebellar neurons from rats and mice to identify the expression profile of TGF-β1	Postnatal: P1, P3, P9, P12, P15, P30	TGF-β1 regulate GCs excitatory synapses and connectivity and express TβRII receptor	[98]

**Table 4:** Summary of reporter and genetic mice models

Mouse model	Description
Col1a1-GFP^GFP/+^	Transgenic reporter line in which GFP expression is driven by Collagen type I alpha 1 promoter
CXCR4 ^−/−^	Full knockout of C-X-C chemokine receptor type 4
Foxc1^−/−^	Knockout of upstream transcription factor of C-X-C motif chemokine ligand 12
Foxc1^hith/hith^	Hypomorphic mutant with ~ 5% Forkhead box C1 activity
GFAP^Cre^;ilk^Flox/Flox^	Conditional knockout of Laminin-β downstream regulator Integrin-Linked Kinase in glia cells
hGFAP^Cre^;Fgfr1^Flox/Flox^;Fgfr2^Flox/Flox^	Double knockout of fibroblast growth factor receptors 1 and 2 in glial progenitors
IGF-1R^KO−tdTomato^	Conditional deletion of IGF-1R in CNS macrophages; used to study IGF signaling in microglia
Igf1r^−/−^:GFP +; Igf1r^+/+^:RFP +	Mosaic model to compare Igf1r null mice and wild-type granule cell precursors in the same environment
IL-33^Gt/Gt (LacZ)^	Gene trap LacZ reporter mouse model; used to study IL-33 expression in cerebellar layers
IL-33^mCherry^;Aldh1l1^eGFP^	Dual reporter for Interleukin-33 and astrocytes; used in synapse maturation studies
IL1RL1^−/−^	Knockout of Interleukin-1 Receptor-Like 1; used to evaluate synaptic pruning deficits
L7^CreERT2^; rRORα1-HA	Inducible Purkinje cell-specific RORα overexpression model; studies impact on Purkinje cells maturation
Math1^Cre^;Igf1r^Flox/Flox^	Conditional knockout of insulin growth factor 1 receptor in granule cell precursors
Nestin^Cre^;Cxcr4Flox^/Flox^	Conditional knockout of C-X-C chemokine receptor type 4 in neural progenitors
Nestin^Cre^;Fgfr2^Flox/Flox^	Conditional knockout of fibroblast growth factor receptor 2 in neural progenitors
Nestin^Cre^;ilk^Flox/Flox^	Conditional knockout of Laminin-β downstream regulator Integrin-Linked Kinase in neural progenitors
Nestin^Cre^;Smad2^Flox/Flox^	Conditional knockout of Smad2 in CNS neural progenitors
Nestin^Cre^;Wnt5a^Flox/Flox^	Conditional knockout of Wnt5a in neural progenitors
Npc1^nmf164^	Genetic mutation in Niemann–Pick disease type C1 gene of microglia model (Model of Niemann-Pick Disease) microglia-specific NPC1
Pcp2^Cre^;Rorα^Flox/Flox^	Conditional knockout of Retinoic acid receptor-related Orphan Receptor Alpha in cerebellar Purkinje cells
Pdgfc^−/−^;Pdgfra^GFP+^	Knockout of Platelet-Derived Growth Factor C with PDGFRÎ ± -GFP (model of cerebellar hypoplasia)
Ptc^±^;Igf1^Tg^	Overexpressing insulin growth factor 1 in neural progenitors (model of medulloblastoma)
Ptch1^±^	Heterozygous knockout of Sonic hedgehog receptor to activate TGF/SMAD3 signaling
RARE-hsp^LacZ^	Transgenic reporter mouse line that expresses LacZ in cells where retinoic acid (RA) signaling is active, enabling spatial and temporal visualization of RA-responsive tissues
SDF-1^−/−^	Full knockout of Stromal Cell-Derived Factor 1
Sox1^Cre^;Cxcr4^Flox/Flox^	Conditional knockout of C-X-C chemokine receptor type 4 in CNS neural precursors
Sox2^Cre+^;Lama1^flox/del^	Conditional knockout of Lama1 in neural precursors
Tmem67^−/−^	Knockout of Wnt5a-associated receptor
γ1III4^−/−^	Knockout of laminin-γ1 domain (γ1III4)

## Humans vs Rodents: Structural differences in the meninges–cerebellum interface

Most of the developmental insights described in this review are derived from rodent models; however, important species-specific differences should be considered when extrapolating to humans. Human meninges are generally thicker and more vascularized, with a denser collagen network in the dura and a more complex venous sinus system compared to rodents (dura thickness: humans ~ 564 µm; rodents ~ 49 µm; blood vessel diameter: humans ~ 45 µm; rodents ~ 20 µm) [50, 51]. Moreover, arachnoid granulations, which CSF drainage, are prominent in humans (~ 200 µm) but are absent in rodents, which instead rely on alternative CSF clearance routes (i.e. glymphatic system) [50]. In terms of timing, meningeal maturation extends into the early post-natal period in rodents, whereas much of this process occurs prenatally in humans. Additionally, immune cell distribution differs between species, with reported variations in the abundance and localization of macrophages, mast cells, and lymphocytes at CNS borders [50]. On the other hand, the human cerebellum is proportionally larger and exhibits a more complex lobular and foliation pattern. Developmental timing also differs as most GCs proliferation and foliation occur postnatally in mice (P0–P21), the majority of these processes take place prenatally in humans, with synaptic refinement continuing into adolescence [31]. Together, these distinctions underscore the importance of cautious extrapolation from rodent models to human meningeal and cerebellar development.

## Meningeal-cerebellar interaction in development

**Fig. 3:**
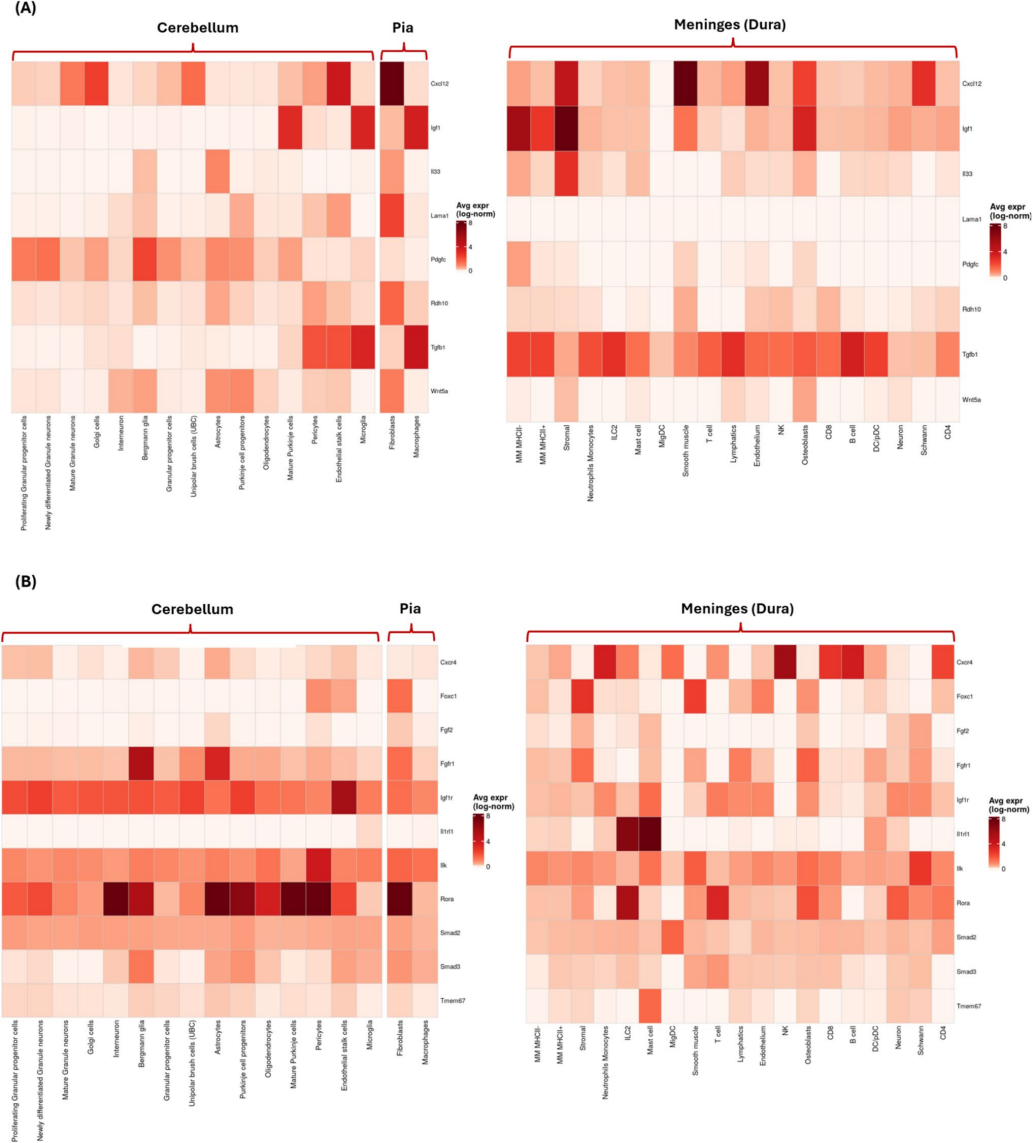
Single-cell expression profiles of ligand–receptor pairs involved in meningeal–cerebellar signaling during early postnatal development. (**A–B**). Heatmaps showing the average normalized expression (log-transformed) of selected ligands (**A**) and their corresponding receptors (**B**) across various cell types in the cerebellum, pia, and dura mater of the meninges, based on published single-cell RNA sequencing data generated by Rejane Rua’s lab [110]. Cell types are grouped by anatomical region, with cerebellar neurons and glial cells (left), pial cells (middle), and dural immune and stromal populations (right). Stronger red intensity indicates higher average gene expression within that cell type. Note that color scales are specific to each dataset and only allow comparison within a dataset, not across datasets. These data highlight potential inter-compartmental signaling axes mediating cerebellar development via meningeal-derived cues

During early postnatal development, meningeal cells attract undifferentiated cells to the pia, and the proper positioning of the EGL depends on stabilizing signals derived from the meninges as shown ex vivo [52]. This allows the establishment of a secondary proliferative zone that is important for shaping the structure and function of the cerebellum during development [52]. A correlative study indicated that intracisternal injection of 6-hydroxydopamine (6-OHDA) in hamsters at different timepoints postnatally led to loss of meningeal cells that disrupted cerebellar growth, caused unorganized foliations and damaged the basal lamina specifically during the first postnatal week [49]. This effect is absent at later timepoints due to the completion of foliations morphogenesis and maturation of glial limitans [49]. Another correlative study conducted by the same group supports this finding where administration of 6-OHDA in rats disrupted meningeal cells leading to significant reduction in cerebellar GCs, abnormal morphology and restricted folia formation [53]. This indicates that meningeal cells prevent ectopic migration of GCPs into the subarachnoid space as they maintain the basal lamina and glia limitans structures located directly underneath the pia matter [46]. As shown in Fig. 1, CD163⁺ meningeal macrophages closely line the pial surface of the developing mouse cerebellum, illustrating the anatomical association between meningeal immune cells and the cerebellum. Overall, these studies highlight the critical role of meningeal cells during key windows of cerebellar development. However, the precise molecular mechanisms by which meningeal cells regulate these processes remain unexplored. To further examine potential interactions between meningeal and cerebellar compartments, we analyzed single-cell RNA sequencing datasets provided by Dr. Rejane Rua lab, profiling the developing meninges (P3) and cerebellum (P0–P8), and visualized the expression of key ligand–receptor pairs involved in cerebellar development and immune signaling (Fig. 3).

### Secreted factors

#### C-X-C motif chemokine ligand 12 (CXCL12)

CXCL12 or Stromal-Derived Factor 1 (SDF-1) is a potent chemoattractant that plays a role in regulating migration and proliferation of hematopoietic precursors [54]. Single cell transcriptomics data using Col1a1-GFP^GFP/+^ reporter mice revealed that CXCL12 is highly expressed in meningeal fibroblasts in the pia starting from embryonic day 14 (E14) [27]. Its receptor CXCR4 is expressed in radial glia end-feet that is anchored at the pia mater regulating radial glia development and function through CXCL12/CXCR4 signaling pathway [55]. In the cerebellum parenchyma, CXCR4 is expressed embryonically at day E13.5 especially in proliferative areas such as the EGL, neuroepithelium and rhombic lip [56]. Absence of CXCR4 in a full knockout mouse model (CXCR4 ^−/−^) led to premature migration of GCPs at embryonic day E17.5, forming ectopic GCPs clusters within the PCL at postnatal day 0 [56]. In addition, CXCR4 ^−/−^ mice exhibited reduced lymphocyte production from the bone marrow, disrupted cerebellum architecture (i.e. irregular EGL and no foliations) at embryonic day E18.5, with a similar phenotype also observed in SDF-1 full knockout mouse model (SDF-1^−/−^) at the same age [57]. A comparative study using CXCR4 ^−/−^ mice support these findings in the context of abnormal GCPs organization at embryonic day E19 without affecting the developmental state of inhibitory interneurons precursors in the EGL as they are independent of the CXCL12/CXCR4 pathway [58]. However, absence of CXCR4 affected pre-cerebellar pathways from the pontine nuclei (PN) as PN neurons had reduced Plexin-D1 (semaphorin-3 receptor) expression that regulates CXCL12/CXCR4 pathway to ensure precise spatial arrangement of migrating neurons [58]. Thus, reduced Plexin-D1 impaired pontine neuron migration and axonal guidance that are critical for forming GC-MF afferents [58]. Furthermore, conditionally knocking-out CXCR4 in neural progenitors (Nestin^Cre^;Cxcr4^LoxP/LoxP^) or deleting SDF-1 upstream transcription factor (Foxc1^−/−^) affected the migratory and proliferative state of neural progenitors such as the GCPs [59]. In addition, Foxc1^−/−^ mice also had reduced radial glia and Bergmann glia cell proliferation, increased cell death and impaired PCs migration from the ventricular zone during embryonic development [59]. It is important to note that radial glia guide PCs migration from the ventricular zone to form the PCL, whereas Bergmann glia acts as scaffolds that guide GCs inward migration from the EGL to the IGL [59]. Moreover, hypomorphic Foxc1^hith/hith^ mice (retaining 5% of Foxc1 activity) exhibited aberrant GCPs migration and disruption in cerebellar foliations and lamination which is an indication of dysregulated mesenchymal signaling embryonically and postnatally [60]. A similar phenotype was observed in cerebellar hypoplasia mouse model (Pdgfc^−/−^;Pdgfra^GFP/+^) lacking platelet-derived growth factor-C (Pdgfc), which shares the same upstream regulator (Foxc1) as the CXCL12/CXCR4 signaling axis [61]. The phenotype exhibited missing lobules and irregular pattern formation of PCs and Bergmann glia at embryonic and postnatal stages [61]. In addition, these mice had downregulation of genes linked to PCs (Calb1) and meninges (CXCL12 and Crym), which is an indication that mice lacking Pdgfc have malformed meninges, altered PCs connectivity and excessive migration of GCPs into the leptomeninges [61]. Additionally, CXCR4 also has a role in cerebellar circuit maturation as transcriptomic profiling of cerebellum from conditional knockout of CXCR4 in CNS neural precursors (Sox1^Cre^;CXCR4^flox/flox^) revealed reduced Neurogenin 2 (Neurog2) and Pancreas specific transcription factor-1a (Ptf1a) which are functionally linked with CXCR4 at postnatal day 1 [62]. This correlates with impaired PCs dendrite formation, dysregulated axon guidance and cell–cell adhesion pathways leading to compromised motor coordination behavior [62]. Together, these findings highlight the essential role of the CXCL12/CXCR4 signaling axis and its upstream and downstream regulators in orchestrating proper cerebellar development through the coordination of meningeal signaling, radial glia organization, neuronal migration, and circuit maturation.

#### Fibroblast growth factor-2 (FGF2)

Fibroblast Growth Factor-2 (FGF2) is a pleiotropic growth factor that is activated through its receptor tyrosine kinases encoding FGFR1-4 [63]. Both the ligand and its receptor are highly expressed in the leptomeninges, especially in endothelial cells and fibroblasts. It acts as a mitogen for meningeal cells and has a role in regulating neuronal differentiation, angiogenesis and proliferation of neural progenitors [64–66]. In the developing cerebellum, FGF2 is markedly upregulated at postnatal day 1, with its expression being precisely regulated in coordination with key developmental milestones, including GCPs in the EGL at P0, GCs in the IGL at P7, and PCs in the PCL by P21 [67, 68]. Furthermore, in situ hybridization revealed that Fgfr1 and Fgfr2 are highly expressed throughout the cerebellum developmental timeline embryonically and postnatally especially in PCs, Bergmann glia cells and GCPs [67]. Absence of both receptors using a double knockout mouse model (hGFAP^Cre^;Fgfr1^f/f^;Fgfr2^f/f^) resulted in abnormal lobule formation and layer morphology, misplaced PCs and reduced molecular layer interneurons, Bergmann glia fibers and GCs proliferation and migration [69]. Conditional knockout of Fgfr2 in neural progenitors (Nestin^Cre^;Fgfr2^flox/flox^) affected cerebellar growth, foliations and disrupted the formation and localization of PCs in the PCL, and disorganized GCPs in the EGL embryonically [70]. Interestingly, lack of Fgfr2 reduced the number and survival rate of Bergmann glia and radial glia precursors that contribute to PCs aberrant positioning and foliation defects [70]. Moreover, FGF-2 promotes the generation of oligodendrocytes from CD44⁺ neural progenitors within neurospheres derived from the cerebellum at postnatal day 3, indicating its role in early postnatal cerebellar development ex vivo [71]. Overall, these findings highlight the critical role of FGF2–FGFR signaling in orchestrating multiple aspects of cerebellar development, including progenitor cell proliferation, laminar organization, and proper positioning of neuronal and glial cell types.

#### Insulin growth factors (IGFs)

Insulin-like Growth Factors (IGFs) play a fundamental role in brain development, contributing to the migration, differentiation, maturation, and survival of neural cells [72]. A comparative study demonstrated that IGF-1, IGF-2, and their receptor IGF-1R are abundantly expressed in various brain regions, including the meninges from early development through maturation in rats [73]. Another study confirmed that BAMs in the meninges locally secrete IGF-1, and conditionally deleting IGF-1R in CNS resident macrophages (i.e. microglia, perivascular and meningeal macrophages) using IGF-1R^KO−tdTomato^ mouse model impairs CNS inflammatory response and transcriptomic profiling of these macrophages, leading to neuroinflammation [74]. In the cerebellum, IGF-1 is expressed in the PCs and its gene expression activity occurs in parallel to the GCs inward migration process, facilitating cerebellar normal development in rats [73, 75]. In situ hybridization supports this finding by identifying the expression patterns of IGF signaling pathway during late embryonic and postnatal cerebellum development (Table 5) [76]. Moreover, IGF signaling modulates Shh pathway, which is primarily driven by PCs acting on GCPs proliferation [76]. These GCPs in addition to Bergmann glial cells express Gli1, a key downstream transcriptional activator of the Shh signaling cascade [76]. Conditional deletion of IGF-1R in GCPs using Math1^Cre^;Igf-1r^flox/flox^ mouse model led to reduced cerebellar weight and GCPs number in the anterior cerebellar foliations while the GCPs in the posterior region remained intact at postnatal day 21 [77]. To support this, the same study created another mouse model to understand how IGF-1R deficient GCPs behave in a normal environment individually (i.e. subset of cells is Igf1r^−/−^;GFP^+^, while the rest are wild-type Igf1r^+/+^; RFP^+^). In this model, the cerebellum had reduced GCs number in both the anterior and posterior cerebellar foliations along with impaired proliferation at postnatal day 21 [77]. This is due to reduced GCPs entry at postnatal day 4 into the S-phase and early cell cycle exit that impaired GCPs proliferation and differentiation leading to a reduced progenitor pool [77]. Interestingly, Insulin-like Growth Factor Binding Proteins (IGFBPs) such as IGFBP-5 inhibit IGF-1/2 action and limit the strength and duration of Shh signaling on GCPs preventing excessive proliferation [76]. Administration of IGF-1 into a denervated cerebellum after unilateral pedunculotomy promoted olivocerebellar reinnervation especially for the PC-CF network, thus creating an environment that supports myelination and neuroplasticity in neonates [78]. Furthermore, overexpression of IGF-1 in neural progenitors in a medulloblastoma mouse model (Ptc^±^;Igf1^Tg^) switched IGF-1 role to favor proliferation over differentiation in the first postnatal weeks promoting brain overgrowth and EGL lesions, leading to the activation of abnormal development pathways that promote metastasis in the cerebellum [79]. Collectively, these findings highlight the multifaceted role of IGF signaling in coordinating cerebellar development, immune cell function, and neural plasticity, while also highlighting its potential contribution to pathological processes when dysregulated.

**Table 5:** Expression pattern of IGF signalling pathway

IGF Signaling	
IGF-1	
IGF-2	Specific to meninges, choroid plexus and cerebellum blood vessels
IGF-1R	Ubiquitously across the cerebellum
IGFBP2	Specific to meninges, choroid plexus, EGL, PCL, IGL
IGFBP3	In the PCL only
IGFBP4	Specific to the meninges and choroid plexus
IGFBP5	Specific in the EGL (proliferating cells) and cells withing the PCL (not PCs)
IGFBP6	

#### Interleukin-33 (IL-33)

Interleukin-33 (IL-33) is part of the IL-1 cytokine family that bind to and activate cells expressing IL1RL1 (Interleukin-1 Receptor-Like 1) [80]. It is highly expressed by mast cells in the meninges and in the developing cerebellum during the first postnatal week [81, 82]. Spatiotemporal expression of IL-33 in the developing mouse brain revealed that IL-33 and IL-33IR are highly expressed in the cerebellum at postnatal day 9 [81]. Moreover, it was observed that IL-33 is localized in cerebellar GCs and white matter layers with minimal expression in the molecular layer in IL-33-LacZ reporter mice (IL-33^Gt/Gt^) [82, 83]. Transcriptomic profiling of a reporter mouse line (IL-33^mCherry^;Aldh1l1^eGFP^) showed IL-33 is highly expressed in astrocytes during synapse maturation at postnatal day 9 in the forebrain [84]. Although its specific role in cerebellar development is not fully understood, IL-33–IL1RL1 signaling between astrocytes and microglia has been shown to promote microglial phagocytic activity, and its absence in the microglia using a full knockout model (IL1rl1^−/−^) impairs synaptic pruning leading to behavioral abnormalities postnatally [85]. Thus, it can be assumed that the IL-33-IL1RL1 pathway has a role in regulating synaptic pruning and myelination in the cerebellum as both are important for proper motor function and neuronal circuit integrity.

#### Retinoic acid (RA)

Retinoic acid (RA) is a metabolic product of vitamin A (retinol) and a lipophilic molecule that mediates critical developmental events associated with neuronal patterning, proliferation and neurite outgrowth. It exerts its effects by binding to and activating two classes of nuclear receptors: retinoic acid receptors (RARs) and retinoid X receptors (RXRs), which in turn regulate gene expression (86, 87). It is synthesized from retinol by a two-step oxidation process involving retinol dehydrogenases (RDH10) and retinaldehyde dehydrogenase (RALDHs) [86]. The developing cerebellum does not produce RA directly, instead it receives it from adjacent structures like the meninges, where meningeal fibroblasts produce several RA signaling genes (i.e. RDH10, ALDH1A2, CRABP2) at embryonic day 14 as part of the development process [27, 88]. Increased RALDH2/RA activity was observed in cerebellar PCs, pre-cerebellar regions such as PN, inferior olive (IO) and the meninges at postnatal day 3 in a reporter mouse line of RA (RARE-hsp-lacZ) [88]. Furthermore, Cellular Retinoic Acid Binding Protein -I (CRABPI) is highly expressed in pontine neurons as it has a role in promoting their migration and inducing differentiation of different cerebellar neuronal groups [88, 89]. Retinoic acid-related orphan receptor α (RORα) is mainly expressed in the PCs and the molecular layer during development, and absence of RORα in PCs using cell-specific knockout mouse model (Pcp2^cre^;Rorα^flox/flox^) affected their morphology (dendrites retraction and spiny branchlets loss) and led to abnormal establishment of CF innervation onto the PCs [90]. Furthermore, knockdown of RORα in the embryonic brain through utero electroporation at embryonic day 11.5 affected PCs dendritic maturation at postnatal day 8, impaired dendritic branches formation at postnatal day 14 and increased PCs axons swelling at postnatal day 21 which is an indication of degenerating neurons [91]. On the contrary, overexpressing RORα in the PCs at postnatal day 4 (L7-CreERT2, 4-OHT–inducible expression of rRORα1-HA) did not enhance dendritic development but instead accelerated PCs maturation process especially at the fusiform stage which would potentially disrupts their synapses formation and circuit development [91]. Interestingly, in a model of ASD where valproic acid (VPA) was injected intraperitoneally at E10.5 in rats, RORα was reduced at the mRNA and protein level in the cerebellum affecting biological processes such as synaptic connectivity in the PCs postnatally, leading to long lasting social behavior deficits in adulthood [92]. Moreover, inducing RA expression through the administration of all-trans RA agonist subcutaneously at postnatal day 0 in rats mainly affected GCs as it reduced their proliferation and prevented their inward migration from the pial surface to the IGL as they remained stuck in the ML [93].These studies highlight the critical role of RA signaling and RORα expression in cerebellar development and how their dysregulation may contribute to neurodevelopmental disorders such as ASD.

#### Transforming growth factor-beta (TGF-β)

Transforming Growth Factor-beta (TGF-β) expression in the meninges is well documented, especially TGF-β1 isoform as it is expressed by meningeal fibroblast in the pia matter [27]. It stimulates different cellular processes including cell proliferation, differentiation and survival, in addition to having a neuroprotective role in the CNS [94–97]. TGF-β1 and TβRII expression profiles are associated with synapses and neural network formation of GCs, especially during the second postnatal week when the cerebellum neuronal circuits develop [98]. Under steady-state, TGF-β1/2 are present in the cerebellar primordium from embryonic day 9 and 13 along with a significant high expression of TGF-β1 at embryonic day 11.5 in the pia mater of wild-type mice embryos [99]. It was also observed in the same embryos that TGF-β1 signaling pathway is associated with regulating developmental and cell–cell interaction pathways (i.e. of β-catenin (CTNNB1) and N-cadherin (CDH2) respectively) during early embryogenesis [99]. Conditional knockout of Smad2 (downstream transcription factor of TGF-β) in CNS neural progenitors (Nestin^Cre^;Smad2^flox/flox^) resulted in irregular cerebellar foliation formation (especially in lobule IX and X), delayed GCs migration and maturation along with impaired PCs dendrites [100]. In addition, these mice exhibited reduced survival rate and motor coordination deficits at postnatal day 18 [100]. Partial knockout of Shh receptor (Ptch1^±^) in mice activated SMAD3 which in turn promotes the progression of medulloblastoma by redirecting certain cerebellar neuronal precursors (i.e. GCPs) to be the origin of metastasis [101]. Thus, it is crucial to regulate TGF-β signaling during cellular development processes [101]. Collectively, these findings indicate the critical yet context-dependent role of TGF-β signaling in cerebellar development, where precise regulation of its downstream mediators, such as SMAD2 and SMAD3, is essential for balancing normal neurodevelopmental processes with the risk of pathological transformation.

#### Wingless-related integration site-5 (Wnt5)

Wnt5a is part of the Wnt family of secreted proteins that are involved in multiple developmental events especially associated with cell–cell communication and patterning during embryogenesis [102]. Meningeal fibroblasts, especially in the arachnoid and pia mater, express several Wnt ligands (e.g. Wnt4, Wnt5a, Wnt5b, Wnt6, Wnt9a and Wnt11) [27]. In the cerebellum, Wnt5a act as a mediator for neural progenitor proliferation and differentiation with a high abundance between embryonic day 18 and postnatal day 3 in mice [103]. Specific deletion of Wnt5a in neural progenitors using a conditional knockout mouse model (Nestin^Cre^;Wnt5a^flox/flox^) led to impaired cerebellar morphology (area, cortical layers and lobulation pattern) and reduced PCs and GCPs progenitors embryonically and postnatally affecting cerebellar progenitors neurogenesis [103]. In addition, by postnatal day 7, Nestin^Cre^;Wnt5a^flox/flox^ mice had restricted PCs growth and branching in addition to reduced EGL proliferation indicating its importance during early development [103]. Interestingly, mice lacking Wnt5a transmembrane protein Tmem67 (Tmem67 ^−/−^) had cerebellar foliation defects and delayed PCs maturations pre- and postnatally [104]. This is associated with reduced expression of Shh downstream protein (Gli1) that is important for PCs maturation and balancing GCPs proliferation/differentiation ratio [104]. Moreover, Tmem67 ^−/−^ mice had dysregulated Wnt/βcatanin signaling, that affected cellular responses to Shh signaling leading to impaired cerebellum development [104]. Together, these findings underscore the essential role of Wnt5a signaling originating from the meninges in orchestrating cerebellar development through its effects on neural progenitor proliferation, PCs maturation, and circuit formation.

### Extracellular matrix laminins

The basal lamina located between the pia matter and glia limitans contains a family of heteromeric extracellular matrix (ECM) proteins consisting of α, β and γ chains known as laminins [105]. Single-cell transcriptomic data showed that meningeal pial fibroblasts at embryonic day 14 had the highest expression of several laminin genes including Lama1, Lama2, Lama4, Lamb1, Lamb2, and Lamc3 that are closely involved in maintaining the meninges structural integrity and brain development [27, 29]. Selective deletion of nidogen binding site of laminin-γ1in mice (γ1III4^−/−^) embryonically affected the pial basement membrane and led to glia cells retraction and ectopic neuron localization outside the meninges, indicating the importance of laminin-γ1 in maintaining an intact pial membrane during development [106]. Interestingly, two studies conditionally knocked out Lama1 in all embryonic tissues derived from the epiblast (Sox2^cre/+^;Lama1^flox/del^) and observed defects in meningeal arrangement especially in the pial basement membrane [105, 107]. This was also accompanied with impaired cerebellar formation and Bergmann glia cells alignment in the absence of Lama1 [105, 107]. However, one study observed a reduction in GCPs proliferation at P0, P5 and P10 while the other reported an increase in proliferation and GCPs apoptosis around the same timepoints [105, 107]. Furthermore, removal of downstream regulator of laminin β Integrin-linked kinase (ILK) using either Gfap^Cre^;ilk^loxP/loxP^ or Nestin^Cre^; ilk^loxP/loxP^ led to defective laminin placement which correlates with loss of cerebellar foliation at postnatal day 10 [108]. In addition, these mice exhibited ectopic GCs positioning into the molecular layer, reduced EGL thickness and altered glia network formation (fewer end-feet in the lamina layer) as a result of the instability of the cerebellum basal lamina [108]. Overall, these studies indicate the critical role of laminin proteins in maintaining the structural and functional integrity of the cerebellar pial basement membrane, which is essential for proper glial scaffold formation, granule cell positioning, and overall cerebellar morphogenesis.

## Perspectives and future work in the field

The emerging role of the meninges as an active contributor to cerebellar development opens exciting avenues for future research. Understanding when specific factors are upregulated, and how they interact with cerebellar cells at different stages from neural progenitor expansion to synaptic refinement will clarify how the timing of meningeal influence shapes cerebellar architecture. Additionally, the spatial specificity of these signals across different meningeal layers (dura, arachnoid, and pia) and cerebellar subregions remain to be mapped in greater resolution using tools like single-cell spatial transcriptomics. Moreover, the interplay between meningeal cells also represents a compelling area for investigation. Emerging studies suggest that meningeal macrophages, B cells, and ILCs not only respond to developmental cues but may actively secrete trophic factors that modulate cerebellar morphogenesis. Determining the precise role of these immune populations under physiological versus inflammatory conditions could reveal novel neuroimmune mechanisms underpinning cerebellar circuit formation. Furthermore, there is growing interest in how external risk factors such as maternal diet or infections impact meningeal signaling and thereby altering cerebellar development. Models such as maternal high-fat diet or prenatal immune activation can be used to understand how extrinsic environmental insults perturb the meningeal-cerebellar axis and contribute to long-term neurodevelopmental disorders, including autism and ataxia. From a translational point of view, characterizing meningeal dysfunction in pediatric cerebellar disorders may provide early biomarkers or therapeutic targets. For example, disruptions in laminin-ILK pathways or RA signaling in the meninges could potentially be modulated to restore normal cerebellar layering and function. Future studies employing inducible and region-specific Cre models, in combination with live imaging and proteomic profiling, will be instrumental in delineating the causal links between meningeal signals and cerebellar development.

Despite that, it is important to highlight that direct causal evidence linking meningeal expression of these factors to cerebellar development remains limited. For many cytokines and growth factors, current data consist primarily of mRNA detection in meningeal tissue. These molecules are also produced by other CNS-resident cells or by circulating immune cells transiently passing through the meninges. Moreover, the BBB may limit the diffusion of meningeal-derived molecules into cerebellar parenchyma, making direct meningeal-cerebellar communication challenging to establish. Although direct evidence is still missing, it is worth noting that the BBB around the cerebellum is more permeable compared to other CNS regions [48, 109]. This regional heterogeneity raises the possibility that certain meningeal-derived factors, particularly during early postnatal stages, may gain access to cerebellar parenchyma, thereby influencing its maturation. Taken together, this review advances a working hypothesis of a bidirectional meningeal–cerebellar interaction. While current evidence remains largely correlative, integrating meningeal biology into the broader framework of cerebellar development offers a more holistic perspective on brain formation. Pursuing this interdisciplinary line of research holds promise for uncovering fundamental developmental mechanisms and informing new therapeutic strategies for cerebellum-linked neurodevelopmental disorders.

## Data Availability

Not applicable.
